# Ultrasound-guided Transvaginal Aspiration in the Management of *Actinomyces* Pelvic Abscess

**DOI:** 10.1155/S1064744996000579

**Published:** 1996

**Authors:** Eyal Y. Anteby, Galiya Rahav, Josef Hanoch, Shlomo Shimonovitz, Simcha Yagel, Neri Laufer

**Affiliations:** 1 Department of Obstetrics and Gynecology Hadassah University Hospital MT. Scopus Jerusalem Israel; 2 Department of Microbiology Hadassah University Hospital Mt. Scopus Jerusalem Israel; 3 Division of Maternal Fetal Medicine Department of Obstetrics and Gynecology Washington University Box 8064, 4911 Barnes Hospital Plaza St. Louis MO 63110 USA

## Abstract

*Background:* Increasing reports of intrauterine device (IUD)-related abdominopelvic actinomycosis have been described recently. Surgical therapy has been the usual treatment when tubo-ovarian abscess is identified.

*Case:* A 38-year-old woman suffering from *Actinomyces* pelvic abscess unresponsive to medical treatment underwent transvaginal ultrasound-guided needle aspiration. It resulted in marked improvement and avoided surgical treatment.

*Conclusion:* Transvaginal needle aspiration of *Actinomyces* pelvic abscess may be an alternative to surgical therapy, thereby allowing the preservation of pelvic organs.


Infectious Diseases in Obstetrics and Gynecology 4:298–300 (1996)
© 1997 Wiley-Liss, Inc.

### Ultrasound-Guided Transvaginal Aspiration in the Management of *Actinomyces* Pelvic Abscess

Eyal Y. Anteby,[1]* Galiya Rahav,[2] Josef Hanoch,[1]
Shlomo Shimonovitz,[1] Simcha Yagel,[1] and Neri Laufer[1]
[1]*Department of Obstetrics and Gynecology Hadassah University Hospital, MT. Scopus, Jerusalem, Israel*
[2]*Department of Microbiology, Hadassah University Hospital, Mt. Scopus, Jerusalem, Israel*

ABSTRACT

***Background:*** **Increasing reports of intrauterine device (IUD)-related abdominopelvic actinomycosis have been described recently. Surgical therapy has been the usual treatment when tubo-ovarian abscess is identified.**

***Case:*** **A 38-year-old woman suffering from *Actinomyces* pelvic abscess unresponsive to medical treatment underwent transvaginal ultrasound-guided needle aspiration. It resulted in marked improvement and avoided surgical treatment.**

***Conclusion:*** **Transvaginal needle aspiration of *Actinomyces* pelvic abscess may be an alternative to surgical therapy, thereby allowing the preservation of pelvic organs. Infect. Dis. Obstet. Gynecol. 4:298–300, 1996.** © 1997 Wiley-Liss, Inc.

KEY WORDS
actinomycosis; abscess; aspiration; ultrasound

Actinomycetes, gram-positive anaerobic or microaerophilic rods, are normal inhabitants of the oropharynx and the bowel.[1] The clinical syndrome of pelvic actinomycosis in association with the "modern" intrauterine device (IUD) was first reported by Henderson.[2] Since then, several case reports have described pelvic actinomycosis in patients with IUDs. The disease rarely occurs unless the IUD has been in place for at least 2 years, and the risk seems to increase with time.[3] The presentation is typically indolent, often delaying the diagnosis. Endometritis and tubo-ovarian abscess are usually the earliest forms of pelvic actinomycosis.[4,5] The disease can spread locally and involve the ureters, bladder, and the rectum.[6] Extension to the abdominal wall, small bowel, and pelvic bones and distant hematogenous dissemination may occur.[7–9] An initial diagnosis of ovarian or uterine tumor is frequently considered, leading to unnecessary extensive surgery. Actinomycotic pelvic abscess is treated initially by large doses of intravenously administered penicillin. Surgical intervention was necessitated in the majority of the cases previously described.

### CASE REPORT

A 38-year-old woman presented to the emergency room with high fever, chills, and lower abdominal pain. Her medical history was unremarkable. She had had 3 normal pregnancies and term deliveries, 2 miscarriages, and 1 induced abortion. She had used a multiload copper IUD for the previous 4 years. For 2 weeks prior to her admission she had suffered from lower abdominal pain. On admission, she had fever of 40°C, pulse 94/min, and blood pressure 120/70 mm Hg. Her abdomen was diffusely tender. On her vaginal examination, the

*Correspondence to: Eyal Y. Anteby, Division of Maternal Fetal Medicine, Department of Obstetrics and Gynecology, Washington University, Box 8064, 4911 Barnes Hospital Plaza, St. Louis, MO 63110.

Received 13 December 1996
Accepted 13 November 1996

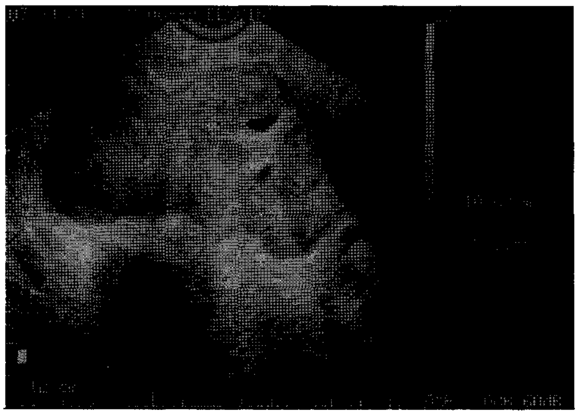

**Fig. 1. Transvaginal sonogram of the actinomycosis pelvic abscesses.**

uterus was extremely tender. A painful mass, 10 cm in diameter, was felt in the pouch of Douglas. The IUD was removed. Pus exuding from the cervix was sent for culture. It showed methicillin-sensitive *Staphylococcus aureus.* Blood and urine cultures were sterile. The erythrocyte sedimentation rate was 150 mm/h, hemoglobin 10.8 g%, and white blood cell count 17,700 with a shift to the left. An ultrasound (US) scan showed an 11 cm, semicystic mass containing both of her ovaries in the pouch of Douglas (Fig. 1). The right ovary contained a 5 x 5 cm hyperechogenic cystic structure consistent with an abscess, and the left ovary contained several loculi, 1–2 cm in diameter. The patient was treated with intravenous amoxicillin-clavulanate (1 g x 3/day), gentamicin (80 mg x 3/day), and doxycycline (100 mg x 2/day). After 5 days of antibiotic treatment the patient remained febrile, with temperatures reaching over 38.5°C. She continued to suffer from abdominal pain and the pelvic and US examination findings were similar to those on her admission. Therefore, a transvaginal US-guided needle aspiration was performed. One hundred twenty milliliters of pus was drained from the large right ovarian abscess and 30 ml from several left ovarian loculi. A Gram stain revealed many polymorphonuclear cells with branching gram-positive filaments. Her culture grew *Actinomyces israelii.* Following the drainage, her fever subsided and the abdominal pain disappeared. She was treated with intravenous penicillin for 2 weeks, followed by oral amoxicillin for 2 months. The pelvic abscess decreased to 6 cm 2 weeks after drainage and finally disappeared.

### DISCUSSION

Pelvic actinomycosis may be a serious, possibly life-threatening disease. Ninety-two cases of actinomycotic pelvic abscesses were reviewed recently by Fiorino.[10] The average age of the patients was 37 years, and the IUDs had been in place for an average of 8 years. The most common complaints were abdominal pain and weight loss. A physical examination was usually uninformative. Uni- or bilateral tubo-ovarian abscesses were present in 90% of the cases, with other organs often involved by the inflammatory process. In the remaining patients, the abscesses involved the bowel, bladder, retroperitoneum, or abdominal wall. Hysterectomy with uni- or bilateral salpingo-oophorectomy was performed in the majority of the patients. One of the major concerns in treating actinomycosis is the possibility of surgical complication, due to the extensive tissue damage and absence of the usual surgical planes, which characterize the disease. Therefore, the treatment of choice is high-dose intravenous penicillin (18–24 million units) for a prolonged time (2–6 weeks), followed by oral therapy for 6–12 months.[10] Yet, in most of the cases, the inability to diagnose the disease preoperatively and the unresponsiveness of the patients to the initial medical treatment lead to high rates of surgical interventions, usually hysterectomy and salpingo-oophorectomy. Fistula formation may also complicate the postoperative course.[10] As a large percentage of women presenting with IUD-related actinomycosis pelvic abscesses are 40 years or younger, this standard surgical treatment can leave them sterile. Reports of prolonged medical therapy alone curing extensive actinomycosis bring the use of ablative surgical therapy into question.[11] Furthermore, percutaneous aspiration or drainage of periappendicular,[12] hepatic,[13] or epigastric[14] actinomycosis has been reported, offering an additional therapy option. This method can also be of value in the diagnosis and treatment of women with actinomycosis pelvic abscess. However, its use in the management of these cases has not been reported.

Transvaginal aspiration of pelvic abscesses and fluid collections caused by other microorganisms has been reported previously.[15–18] In these cases, the diagnosis of pelvic abscess was made by the characteristic symptoms and physical examination, combined with the appearance of a complex cystic mass on transvaginal US. Transvaginal needle aspiration yielded 35–150 ml of purulent fluid, with positive bacteriologic cultures being observed in 60–80% of the patients. *Escherichia coli* was the isolated microorganism in the majority of the patients. Other bacteria included *Klebsiella, S. aureus, Streptococcus* α *hemolyticus,* and *Acinetobacter.* Complete evacuation of the abscesses was usually achieved. No complications were related to the procedure, and full clinical improvement was reported by all authors.

In the presented case, transvaginal US-guided needle aspiration combined with antibiotic treatment was a safe and simple method of treating actinomycosis pelvic abscess. We suggest that when a patient with IUD-associated pelvic actinomycosis does not respond to medical therapy, transvaginal US-guided aspiration should be considered. Surgical treatment can be reserved for those cases who do not improve following the transvaginal drainage.

### REFERENCES

1. Evans DTP: *Actinomyces israelii* in the female genital tract: A review. Genitourin Med 69:54–59, 1993.
2. Henderson SR: Pelvic actinomycosis associated with an intrauterine device. Obstet Gynecol 41:726–732, 1973.
3. Chatwani A, Amin-Hanjani S: Incidense of actinomycosis associated with intrauterine device. J Reprod Med 39(8):585–587, 1994.
4. Burkman R, Schlesselman S, McCafrrey L, et al.: The relation of genital tract actinomycetes and the development of pelvic inflammatory disease. Am J Obstet Gynecol 143:585–589, 1982.
5. Anteby E, Milwidsky A, Goshen R, Ben-Chetrit A, Ron M: IUD associated abdominopelvic actinomycosis. Harefua 121:150–153, 1991.
6. Muller Hozner E, Ruth NR, Abfalter E, et al.: IUD associated pelvic actinomycosis: A report of 5 cases. Int J Gynecol Pathol 14(1):70–74, 1995.
7. Shurbaji MS, Gupta PK, Newman MM: Hepatic actinomycosis diagnosed by fine needle aspiration. Acta Cytol 31:751–755, 1982.
8. de la Monte SM, Gupta PK, White CL III: Systemic *Actinomyces* infection. A potential complication of intrauterine contraceptive device. JAMA 248:1876–1877, 1982.
9. McBride WJ, Hill DR, Gordon DL: Chest wall actinomycosis in association with the use of an intrauterine device. Aust NZJ Surg 65(2):141–143, 1995.
10. Fiorino AS: Intrauterine contraceptive device-associated actinomycotic abscess and *Actinomyces* detection on cervical smear. Obstet Gynecol 87:142–149, 1996.
11. Schleck W, Gelfand M, Alper B, et al.: medical management of visceral actinomycosis. South Med J 76:921–922, 1983.
12. Goldway S, Abbitt P, Wattf B: Case report: Percutaneous drainage of periappendiceal actinomycosis. Clin Radiol 44:422–424, 1991.
13. Grander JK, Houn H-YD: Diagnosis of hepatic actinomycosis by fine-needle aspiration. Diagn Cytopathol 7: 95–97, 1991.
14. O'Connor KF, Bagg MN, Croley MR, Schabel SI: Pelvic actinomycosis associated with intrauterine devices. Radiology 170:559–560, 1989.
15. Aboulghar MA, Mansour RT, Serour GI, Sattat MA, Awad MM, Amin Y: Transvaginal ultrasonic needle guided aspiration of pelvic inflammatory cystic masses before ovulation induction for in vitro fertilization. Fertil Steril 53:311–314, 1990.
16. Aboulghar MA, Mansour RT, Serour GI: Ultrasonographically guided transvaginal aspiration of tubo-ovarian abscesses and pyosalpinges: An optional treatment for acute pelvic inflammatory disease. Am J Obstet Gynecol 172:1501–1503, 1995.
17. Teisala K, Heinonen PK, Punnonen R: Transvaginal ultrasound in the diagnosis and treatment of tubo-ovarian abscess. Br J Obstet Gynaecol 97:178–180, 1990.
18. Van Sonnenverg E, D'Agustino HB, Casola G, et al.: US guided transvaginal drainage of pelvic abscesses and fluid collections. Radiology 181:53–56, 1991.
